# Adult-onset renal cell carcinoma associated with Xp11.2 translocation/TFE3 gene fusion: 3 case reports and review of literature

**DOI:** 10.1097/MD.0000000000011023

**Published:** 2018-06-15

**Authors:** Pengfeng Gong, Qianfeng Zhuang, Kun Wang, Renfang Xu, Yiming Chen, Xiaogang Wang, Shuai Yin

**Affiliations:** Department of Urology Surgery, The First People's Hospital of Changzhou, Changzhou, China.

**Keywords:** imaging, immunohistochemistry, renal cell carcinoma, TFE3, Xp11.2 translocation

## Abstract

**Rationale:**

Renal cell carcinoma associated with Xp11.2 translocations/TFE3 gene fusions is a rare subtype of renal cell carcinoma. This predominantly occurs in juveniles, but rarely seen in adults with lymph node or organic metastasis and a worsened prognosis.

**Patients concerns:**

Herein, we presented 3 adult cases of Xp11-RCC. Two patients were in early stage and good condition, and the third patient had lymph node metastasis but showed no recurrence after a 3-month follow-up.

**Diagnoses:**

Case 1: A 50-year-old female without any lumbago and gross hematuria was incidentally detected by left renal mass by ultrasonography. Case 2: A 31-year-old female with 2-year hemodialysis was detected with right renal carcinoma during preoperative examination of renal transplant. Case 3: A 45-year-old male with right lumbago for 1 month was detected with a mass in the lower pole of right kidney by ultrasonography.

**Intervention:**

The characteristics of these 3 images are not consistent with each other, and showed some differences with the previous ones.

**Outcomes:**

All these 3 patients underwent laparoscopic radical nephrectomy, and case 1 patient underwent renal hilar lymphnode dissection at the same time. Immunohistochemistry was performed on all the 3 tumors, revealing that the tumor cells were positive for TFE3 and Melan-A. Case 1 showed lymph node metastasis, and received mTOR inhibitors. The 3 patients had no recurrent and new metastasis in other organs after follow-up for 3 months, 2 months, and 11 months, respectively.

**Lessons:**

Whether the adult-onset Xp-RCC has an aggressive clinical course still remains controversial. Characteristics of the images of the 3 adult cases showed some uniformity but still have some differences. Immunohistochemistry results revealed tumor cell positive for TFE3, but have no consistency in carbonic anhydrase IX, CD117, Ki67, CK8/18 AE1/AE3 and so on. Therefore, the uniform and definitive diagnostic standards of the tumors are uncertain. Hence, more cases and findings are required to elaborate the standards of all the tumor subtypes. Vascular endothelial growth factor-targeted therapy showed some efficacious results in patients with metastasis, but more useful treatments are warranted.

## 1 Introduction

Renal cell carcinoma associated with Xp11.2 translocation/TFE3 gene fusion is a rare and new subtype of RCC, and is classified by WHO in 2004.^[1]^ This tumor frequently occurs in children and young people, and rarely occurs in middle-aged and old people.^[2]^ Older patients with lymph nodes metastasis usually have worsened prognosis.^[2]^ Herein, we reported 3 cases with Xp11.2-RCC.

## 2 Case presentation

The study protocol was approved by the Ethics Committees of The First People's Hospital of Changzhou, and all participants provided written informed consent.

### 2.1 Case 1

**Figure 1:**
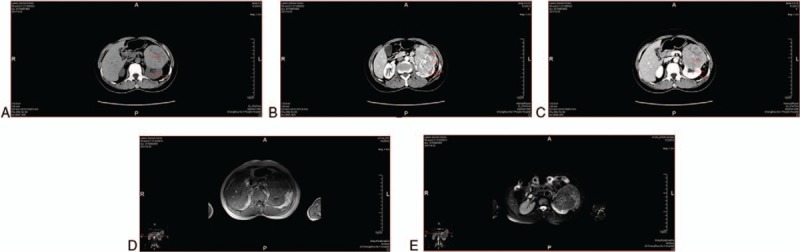
(A) A mixed density mass with a size of 7.9 × 7.6 cm and clear boundary in the lower left kidney were observed. Tumor attenuation (68 HU) was greater than renal parenchyma (33 HU) and medulla (28 HU) during CT plain scan. (B) and (C) There are slightly high and low density necrosis in the inferior plane of the tumor, revealing obvious uneven enhancement and the phenomenon contrast agent fast forward and fast out. The left renal pelvis oppressed by the tumor was unclear. After the peritoneum, the nodular shadow was seen in the retroperitoneal region, showing obvious inhomogeneous enhancement. The attenuation (98 HU in the arterial phase, 101 HU in the venous phase) was weaker than renal parenchyma (180 HU in the arterial phase, 202 HU in the venous phase) and medulla (142 HU in the arterial phase, 125 HU in the venous phase) in both arterial and venous phases. (D) and (E) The left kidney was with an irregular contour. In the upper left pole of the kidney, there were a mass of about 10.6 × 7.9 cm. This was mixed with a short T1 signal, and the internal signal was uneven. The vascular shadows and false envelop was observed in the tumor. Enlarged lymph nodes of about 2.3 cm were observed in the left renal hilar region. On MRI, the signal of the tumor was close to the renal cortex in the T1 and low signal intensity in T2.

A 50-year-old healthy female previously revealed the presence of a left renal mass by ultrasound 3 weeks ago. The patient had no significant back pain and gross hematuria. The abdominal contrast-enhanced CT demonstrated the presence of a mixed density mass with a size of 7.9 × 7.6 cm and clear boundary in the upper pole of left kidney (Fig. 1A). A slightly high- and low-density necrosis was found in the interior of the tumor, revealing an obvious uneven enhancement and the phenomenon of contrast agent fast forward and fast out (Fig. 1B and C). The left renal pelvis oppressed by the tumor was unclear. After the peritoneum, the nodular shadow was seen in retroperitoneal region, showing an obvious inhomogeneous enhancement (Fig. 1B and C). MRI revealed that the left kidney had an irregular contour. A mass of about 10.6 × 7.9 cm was observed in the upper left pole of the kidney. This was mixed with a short T1 signal, and showed an uneven internal signal. The vascular shadows and false envelop can be seen in the tumor. The enlarged lymph nodes were observed in the left renal hilum, with a diameter of about 2.3 cm (Fig. 1D and E). The patient under general anesthesia underwent radical resection of left renal carcinoma and renal hilar lymph node dissection through retroperitoneal route and resected the perirenal fascia, perirenal fat, kidney, ipsilateral adrenal, ureter above the iliac blood vessel bifurcation, and abdominal aorta and inferior vena cava lymph node from the angle of diaphragm to the bifurcation of the abdominal aorta. After incision of the kidney, a cut surface rotten bleeding of gray white tumor of 9 × 8 × 8 cm, and atrophied renal parenchyma were observed. Pathological examination revealed that the case was considered to be XP11.2 tanslocations/TFE3 gene fusions associated renal cell carcinoma with a size of 9× 8 × 8 cm, vascular region showed invasion of carcinoma, and left renal hilar lymph nodes were with metastases (2/2). PET-CT was performed, which revealed no other lymph nodes and organic metastases. So, the tumor observed was in T2N2M0 stage and IV stage according to AJCC Cancer staging Manual. Immunohistochemistry results revealed positive for CAIX, CD117, Ki67, Melan-A, TFE3 (+), AE1 / AE3 and CK8/18 (+), and negative for CD10, CK7, HMB, P504 s, Vimentin, EMA, PAX-8, and SMA. After operation, the patient was given sorafenib 400 mg bid. After 3 months follow-up, the patient was in good condition.

### 2.2 Case 2

**Figure 2:**
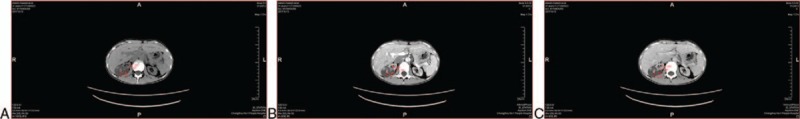
(A) A solid cystic cystic and low density foci in the right kidney, and a nodular and slightly high density shadow within the foci were observed. The tumor contained the cystic part and solid part. During the CT plain scan, the solid part attenuation (53 HU) was greater than the cortex (34 HU) and medulla (32 HU), and the cystic part attenuation (16 HU) was smaller than the cortex (34 HU) and medulla (32 HU). (B) and (C) In the arterial and venous phases, the cystic part (26 HU in the arterial phase, 25 HU in the venous phase) of the tumor showed no enhancement, while the solid part of the attenuation (97 HU in the arterial phase, 83HU in the venous phase) was larger than the medulla (50 HU in the arterial phase, 68 HU in the venous), but lesser than the cortex (103HU in the arterial phase, 90 HU in the venous phase).

A 31-year-old female, who was not married, has been undergoing hemodialysis for more than 2 years without any obvious symptoms underwent CT for preparation of kidney transplantation. CT showed a solid cystic and low-density foci in the right kidney, and a nodular and slightly high-density shadow within the foci (Fig. 2A). The patient underwent radical resection of right renal carcinoma under general anesthesia. After the operation, the kidney was opened, and a white mass of 4 cm size and fine papilla on the surface of the lump in the right kidney was observed. Postoperative pathology revealed Xp11.2 translocations/TFE3 gene fusions associated with renal cell carcinoma with a size of 4∗3 cm. Immunohistochemical results showed positive for AE1/AE3, CD10, Vimentin, CD117, P504 s, Melan-A, and TFE3. The patient did not receive any other treatments. She was followed up for one month after operation, and showed no recurrence.

### 2.3 Case 3

**Figure 3:**
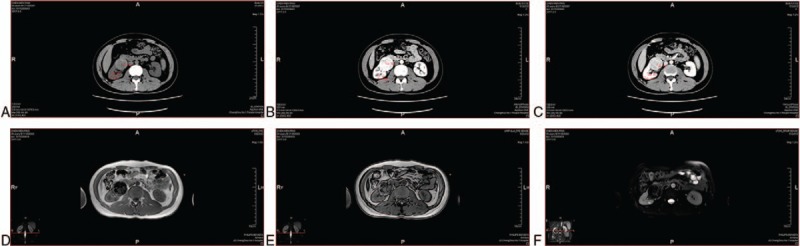
(A) A 5.2 × 4.9 cm round tumor, with clear boundary and uneven density was observed. The tumor attenuation was greater than that of the renal parenchyma and medulla. (B) and (C) In the arterial phase, the tumor enhancement (142 HU) was greater than the medulla (71HU) and lesser than the cortex (233 HU). In the venous phase, the tumor enhancement (116 HU) was weaker than the medulla (148 HU) and cortex (172 HU). (D), (E) and (F) The tumor showed low signal intensity in T1WI, lipid signal out of phase, and long signal in T2WI.

A45-year-old male, with a right lumbago for 1 month, underwent B mode ultrasonography. Results revealed a lower echo mass in the lower pole of the right kidney. The tumor was with a size of 5.4 × 4.8 cm, had a regular shape, but showed no clear boundary, and the internal echo was uneven. Then he was admitted in the hospital, and underwent middle abdomen plain and enhanced CT. Round tumor of 5.2 × 4.9 cm round tumor, with clear boundary and uneven density was observed (Fig. 3A). Laparoscopic radical nephrectomy was performed to open the right kidney. The right renal tumor with pigmentation had a clear tumor boundary. The tumor was considered as translocation of XP11.2 tumor associated with pigment differentiation, and had a low malignant biological behavior. Immunohistochemistry revealed positive for HMB45, Melan-A, S100, Ki67, and TFE3. After 3 months of operation, the patient showed no recurrence and did not undergo any other treatment.

## 3 Discussion

XP11.2 translocation /TFE3 gene fusion associated renal cell carcinoma showed a very low incidence. It mainly occurs in children and young people, accounting for about 20% to 40%^[2]^ of renal cell carcinoma in children. While the proportion of renal cell carcinoma in adults was only 1% to 1.6%.^[2]^ According to the reports of Argani et al, Xp11.2-RCC resulted from the fusion of TFE3 gene with 1 of 5 different genes, including ASPL (17q25), PRCC (1q21), PSF (1q34), NonO (Xq12) and CLTC (17q23).^[3,4]^ The main clinical manifestations of this type of renal cell carcinoma include hematuria and lumbago, and also frequently occur in the clear cell renal cell carcinoma patients. According to the previous reports, the morphology of this tumor was different from that of the clear cell renal cell carcinoma.^[5]^ It is a solid cystic mass that is located in the cortex and often accompanied by internal hemorrhage and peritumor calcification, and has a clear boundary with surrounding tissues.^[6]^ Patients 1 and 3 had a well-defined solid mass, with hemorrhage and no rim calcification. Patient 2 had a solid cystic mass, which may be associated with her hemodialysis for more than 2 years.

The renal cell carcinomas have their own characteristic upon imaging. Kato et al^[7]^ reported that the imaging findings of XP11.2-RCC may be similar to those of papillary RCC in the existence of morphological overlap between the tumor and Papillary RCC. Previous scholars summed up the CT and MRI reports of 20 cases ^[8]^, which showed tumour attenuation in the CT plain in vast majority of cases are stronger than that in renal parenchyma. But during all enhanced phases, tumor enhancement remained weaker than the renal cortex, but greater than the medulla.^[8]^ On delayed phase, the tumor was weaker than the medulla. On MRI, the tumor remained at the same intensity in T1WI, low signal intensity in T2WI was uneven, and slightly high signal intensity in DWI.^[8]^ Liu et al^[9]^ summarized the CT and MRI reports of 5 patients. MRI of 4 cases showed moderate intensity, high signal intensity on T1-weighted MRI, 3 cases showed low signal intensity on T2-weighted imaging, and the other patient showed a relatively high signal intensity because of tumor hemorrhage. CT of 2 cases showed slightly higher tumor density than that of the cortex.^[9]^ CT results of these 3 cases are not consistent with each other, and are not exactly the same as those of the above results. In case 1, the tumor attenuation was stronger than the renal parenchyma and medulla during CT plain scan (Fig. 1A). The attenuation, without including the hemorrhage part, was weaker than renal parenchyma and medulla in both arterial and venous phases (Fig. 1B and C). On MRI, the signal intensity of the tumor was slightly shorter hybrid than that of the renal cortex in T1 and hybrid signal intensity in T2 (Fig. 1D and E). On MRI enhancement, the tumor had uneven enhancement, and vascular shadow and pseudocapsule (Fig. 1D and E). On CT of case 2, the tumor contained cystic part and solid part. During the CT plain scan, the solid part attenuation was greater than the cortex and medulla, and the cystic part attenuation was smaller than the cortex and medulla (Fig. 2A). In the arterial and venous phases, the cystic part of the tumor was not enhanced, while the solid part of attenuation was larger than the medulla, but lesser than the cortex (Fig. 2B and C). On CT scan of case 3, tumor attenuation was greater than that of the renal parenchyma and medulla (Fig. 3A). In the arterial phase, tumor enhancement was greater than the medulla and lesser than the cortex (Fig. 3B). In the venous phase, the tumor enhancement was weaker than the medulla and cortex (Fig. 3C). On MRI, the tumor showed hybrid signal intensity on T1WI (Fig. 3D), lipid signal out of phase (Fig. 3E), and low-signal intensity in T2WI (Fig. 3F). The imaging characteristics of CT and MRI of case 3 are almost consistent with that of the previous cases. However, the tumor in case 1 was in the advanced stage, and had hemorrhage and necrosis. So, the imaging results are clearly different from the other reported cases. The patient in case 2 had hemodialysis history for 2 years and so the tumor had became a solid cystic mass, showing a particular imaging result. Compared to our study cases, the main CT characteristics of XP-RCC include a solid mass in the cases reported previously. Of which, the tumor attenuation was greater than that of the renal parenchyma and medulla in CT plain scan. This was between the cortex and medulla in the arterial phase, which was weaker than the medulla and cortex in the venous phase. Whereas the enhancement was completely different if the tumor has necrosis and hemorrhage inside. On MRI, the tumor showed a intensity mass and a higer signal in T1WI, and a lower signal intensity in T2WI. However, if the tumor involves hemorrhage and lipid, the signal can be hybrid both in T1WI and T2WI. Necrosis and hemorrhage of tumor frequently occurs in the advanced stage patients, and the tumor stage may be related to the age and sex of the patient. Based on these main imaging characteristics, more cases need to be collected to improve the imaging conclusion.

**Figure 4:**
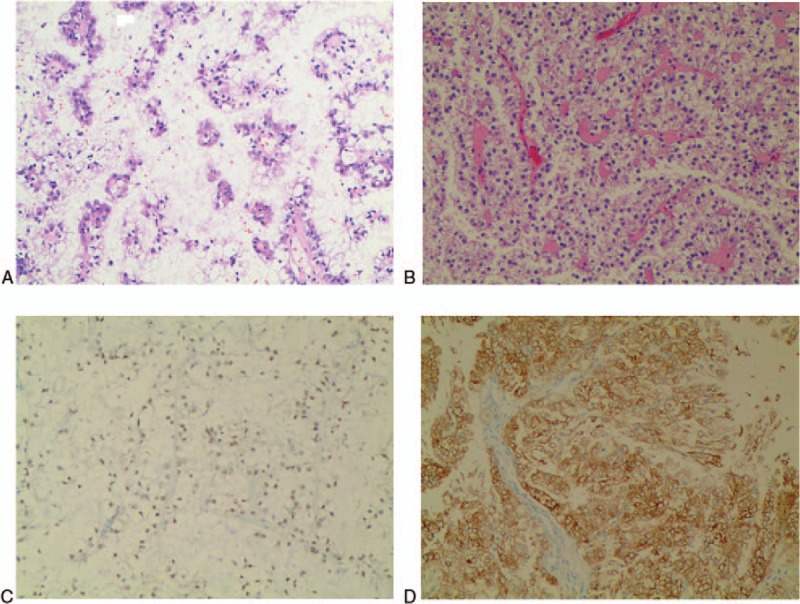
(A) Papillary cell arrangement, abundant cytoplasm, eosinophilic, hyaline nodules and psammoma bodies were observed. (B) The cystic part was encapsulated with friable mural nodules; (C) Positive cytoplasmic staining of TFE3; (D) Positive cytoplasmic staining of Melan-A.

Xp11.2 translocation /TFE3 gene fusion associated renal cell carcinoma is usually diagnosed by pathological examination. This is characterized by papillary cell arrangement, abundant cytoplasm, eosinophilic, hyaline nodules, and psammoma bodies.^[10]^ All the 3 cases reported by us showed similar results as mentioned above microscopically (Fig. 4A). Case 2 tumor had cystic part, which was encapsulated with friable mural nodules (Fig. 4B). All the cases reported in the previous literature have common points, both were TFE3 and CD10 are positive. While the 5 cases reported by Meyer et al^[11]^ showed Vimentin positive, but SMA, CD45 and HMB45 were negative. In one case reported by Henry,^[10]^ AE1/AE3, CK7, EMA, RCA, and CAIX were all positive. Of the two cases reported by Ahluwalia,^[12]^ CD10, Vimentin and EMA were positive, while CK7 was negative. As shown in Table 1, immunization of both TFE3 (Fig. 4C) and Melan-A (Fig. 4D) were positive. In case 1, carbonic anhydrase IX, CD117, Ki67, CK8/18 and AE1/AE3 were positive. In patient 2, CD117, Vimentin, CD10, P504s, and AE1/AE3 were all positive. In patient 3, Ki67, S100, and HMB45 were all positive. Therefore, as shown in Table 1, the immunohistochemical results of the combined case reports of the tumor are not consistent. Although the immunohistochemical results, except TFE3, was little helpful for the diagnosis of this tumor, we speculated that the immunization may be associcated with sex, age, tumor morphology, size, stage, and prognosis. Further exploration for the question was still needed. The first choice for the treatment of renal cell carcinoma associated with Xp11.2 translocation/TFE3 gene fusion include radical nephrectomy, and renal hilar lymph node dissection should be performed at the same time if the patients have lymph node metastases. However, as for localized Xp11.2-RCC, there are no references till date reporting this information. For patients with lymph nodes and/or other distant organ metastases, cytokine therapy including IL-2 and IFN-α and vascular endothelial growth factor targeted therapy (such as sunitinib, sorafenib and monoclonal anti-VEGF antibodies) are required.^[13,14]^ Choueiri et al^[14]^ divided 15 patients with adult metastatic Xp 11.2 translocation renal cell carcinoma into 3 groups. Of which, 10, 3, and 2 patients received sunitinib, sorafenib, and monoclonal anti-VEGF antibody, respectively. Three patients had partial response, 7 had stable disease and 5 had progressive disease. The median OS of all the patients were 14.3 months.^[13]^ Case 1 patient received sorafenib after the surgery, and showed no recurrence after 3 month follow-up. So, VEGF-targeted therapy showed some efficacy in adult patients of Xp11.2-RCC with lymph node or organic metastasis.

**Table 1:**
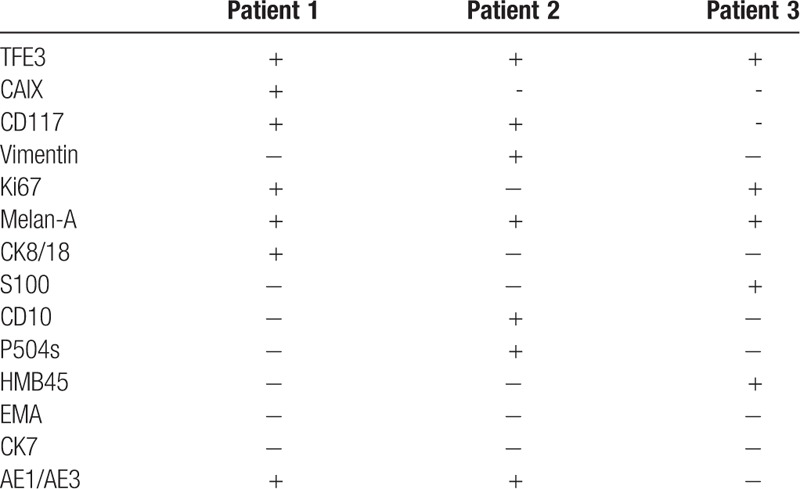
The immunostain profiles of 3 patients.

Xp11.2-RCC mainly occurs in young individuals, and recent studies demonstrated that the prognosis of children was better than that of the adults.^[15,16]^ Kuroda et al ^[17]^ thought that the tumors of children and young adults at advanced stage, including lymph node metastasis, were indolent. According to a case report in 2015, a 17-year-old boy with Xp 11.2 translocation associated with RCC as a second tumor had a long-term survival rate.^[18]^ But as shown by a systematic review and meta-analysis of observational studies,^[19]^ no significant differences were observed in the prognosis between children and adults, and between female and male. Qiu et al^[20]^ and Klatte et al^[21]^ demonstrated that Xp11.2-RCC patients has poorer prognosis whatever treatment was applied. Therefore, there is a dispute whether age and sex have an impact on the prognosis of the tumor. According to several other previous reports, positive expression of TFE3 was associated with clinical paremeters and its prognosis.^[20–22]^ As reported by Mir et al,^[22]^ 7 of 8 TFE3-positive patients had lymph node metastasis, whereas 5.8% of TFÊ3-negative patients had lymph node metastasis and cava thrombus. At the laboratory of Klatte et al,^[21]^ high TFE3 expression was associated with lymph and organic metastasis, which showed poor prognosis.^[21]^ The adult female patient in case 1 had renal hilar lymph node metastasis, and received sorafenib 400 mg bid for 3months. Results showed no significant distant metastasis. Case 2 and 3 patients showed no metastasis in other organs, and did not undergo any other treatments. Therefore, the prognosis of this tumor was affected by tumor stage, which may be associated with TFE3 expression. The prognosis was affected by many factors, such as age, sex or other immunohistochemical parameters, like CD10, Viminten and so on. These were associated with the prognosis, but cannot determine the prognosis lonely.

Herein, we presented 3 cases of adult RCCs associated with Xp11.2 translocations, and investigated the common characteristics of the tumors with the help of imaging. However, if the patients received hemodialysis or the tumor had necrosis and hemorrhage, the imaging results might differ. The main treatment for localized Xp11.2 RCC was radical resection of renal carcinoma, and for advanced renal cell carcinoma with lymph node metastasis, radical resection of renal carcinoma and renal hilar lymph node dissection are essential. The VEGF-targeted therapy showed some efficacious results in the adult patients of Xp11.2 RCC with lymph node or organic metastasis. After the surgery, immunization including TFE3 expression was necessary, indicating the prognosis of the patient. Detection of the relationship between immunization and clinical parameters needs more cases and research.

## Author contributions

**Data curation:** Pengfeng Gong.

**Formal analysis:** Pengfeng Gong.

**Investigation:** Pengfeng Gong, Qianfeng Zhuang, Kun Wang, Renfang Xu, Yiming Chen, Xiaogang Wang, Shuai Yin.

**Methodology:** Pengfeng Gong, Qianfeng Zhuang, Kun Wang, Renfang Xu, Yiming Chen, Xiaogang Wang, Shuai Yin.

**Validation:** Pengfeng Gong, Qianfeng Zhuang, Kun Wang.

**Visualization:** Pengfeng Gong, Qianfeng Zhuang, Kun Wang.

**Writing – original draft:** Pengfeng Gong, Qianfeng Zhuang, Kun Wang, Renfang Xu, Yiming Chen, Xiaogang Wang, Shuai Yin.

**Writing – review & editing:** Pengfeng Gong, Qianfeng Zhuang, Kun Wang, Renfang Xu, Yiming Chen, Xiaogang Wang, Shuai Yin.
